# Myeloid-Derived Suppressor Cells in COVID-19: The Paradox of Good

**DOI:** 10.3389/fimmu.2022.842949

**Published:** 2022-04-27

**Authors:** Germana Grassi, Stefania Notari, Simona Gili, Veronica Bordoni, Rita Casetti, Eleonora Cimini, Eleonora Tartaglia, Davide Mariotti, Chiara Agrati, Alessandra Sacchi

**Affiliations:** Laboratory of Cellular Immunology and Pharmacology, National Institute for infectious Diseases “Lazzaro Spallanzani”—Istituto di Ricovero e Cura a Carattere Scientifico, Rome, Italy

**Keywords:** PMN-MDSC, M-MDSC, SARS-CoV-2, COVID-19, immune response

## Abstract

Severe acute respiratory syndrome coronavirus 2 (SARS-CoV-2) is the causative agent of the ongoing coronavirus disease 2019 (COVID-19) pandemic. Viral replication in the respiratory tract induces the death of infected cells and the release of pathogen- associated molecular patterns (PAMPs). PAMPs give rise to local inflammation, increasing the secretion of pro- inflammatory cytokines and chemokines, which attract immune cells from the blood into the infected lung. In most individuals, lung-recruited cells clear the infection, and the immune response retreats. However, in some cases, a dysfunctional immune response occurs, which triggers a cytokine storm in the lung, leading to acute respiratory distress syndrome (ARDS). Severe COVID-19 is characterized by an impaired innate and adaptive immune response and by a massive expansion of myeloid-derived suppressor cells (MDSCs). MDSCs function as protective regulators of the immune response, protecting the host from over-immunoreactivity and hyper-inflammation. However, under certain conditions, such as chronic inflammation and cancer, MDSCs could exert a detrimental role. Accordingly, the early expansion of MDSCs in COVID-19 is able to predict the fatal outcome of the infection. Here, we review recent data on MDSCs during COVID-19, discussing how they can influence the course of the disease and whether they could be considered as biomarker and possible targets for new therapeutic approaches.

## Introduction

Severe acute respiratory syndrome coronavirus 2 (SARS-CoV-2) is the causative agent of the ongoing coronavirus disease 2019 (COVID-19) pandemic. It is a positive-sense, single-stranded RNA virus of the Coronaviridae family. The pathogenesis of SARS-CoV-2 initiates when the viral particles infect airway epithelial cells, alveolar epithelial cells, vascular endothelial cells, and macrophages in the lung through angiotensin-converting enzyme 2 (ACE2) (1). Viral replication induces the cell destruction and the release of pathogen-associated molecular patterns (PAMPs), inducing local inflammation characterized by increased secretion of the pro-inflammatory cytokines and chemokines interleukin 6 (IL-6), interferon gamma (IFN-γ), monocyte chemoattractant protein 1 (MCP1), and IFN-γ-inducible protein 10 (IP-10) (2). These cytokines and chemokines attract immune cells, notably monocytes and T lymphocytes, from the blood into the infected lung (3).

In most individuals, lung-recruited immune cells clear the infection, and the immune response retreats. However, in some cases, a dysfunctional immune response occurs, which triggers a cytokine storm, mediating widespread lung inflammation. In particular, IL-6 was associated with a high risk of mortality during COVID-19 (4). IL-6 can stimulate various cell types expressing the membrane-bound IL-6 receptor and the glycoprotein (gp130) receptor, leading to constitutive activation of the Janus kinase (JAK) signal transducer and activator of transcription (STAT) (5, 6). Accordingly, JAK-STAT signaling has been shown to be increased in COVID-19 patients (7, 8). STAT3 activation promotes IL-6 gene expression, amplifying this inflammatory pathway (6). On the other hand, the suppressor of cytokine signaling 3 (SOCS3) pathway, which has negative feedback on IL-6 production, is downregulated during COVID-19, thus contributing to IL-6 hyper-production (9). In turn, the uncontrolled inflammation can cause multi-organ damage, leading to organ failure. A large body of literature reported the effects of immune-based therapies targeting inflammatory mediators such as JAK or cytokine inhibitors, demonstrating improved outcomes and survival (10, 11).

Beyond cytokine storm, lymphopenia, high leukocyte counts, and increased neutrophil–lymphocyte ratios have been described as features of severe COVID-19 (12). Neutrophils have been demonstrated to play a role in COVID-19 pathology through the release of neutrophil extracellular traps (NETs). Indeed, tracheal aspirates and pulmonary autopsies from COVID-19 patients showed NET-containing microthrombi` and neutrophil–platelet infiltration (13, 14). SARS-CoV-2 can directly induce the release of NETs by healthy neutrophils, and NETs released by SARS-CoV-2-activated neutrophils promote lung epithelial cell death *in vitro* (14).

In order to dissect the biology of the virus–immune system interaction during COVID-19, several studies employed high-dimensional phenotypic and molecular approaches. These studies highlighted the absence of an IFN signature in severe patients compared to mild and moderate cases and evidenced a sustained emergency myelopoiesis associated with an increase in immature neutrophils and monocytes with immunosuppressive features (15–18). Among immunosuppressive cells, myeloid-derived suppressor cells (MDSCs) emerged as one of the players in the pathogenesis of SARS-CoV-2 infection.

Herein, we review recent data on MDSCs during COVID-19 and their pleiotropic activities, discuss how they can influence the course of the disease, and whether they could be considered as possible targets for new therapeutic approaches.

## MDSC Differentiation

A huge number of leukocytes are generated and replaced daily. Different pathological conditions can perturb the leukocyte turnover, resulting in the emergency of myelopoiesis (19) to provide cells for eliminating tumor cells, infectious agents, or tissue damage. If these conditions resolve quickly, the myelopoiesis declines without negative consequences for the host. However, a number of conditions associated with various types of chronic inflammation result in aberrant sustained myelopoiesis characterized by the accumulation of immature myeloid cells with regulatory functions, ultimately defined as MDSCs (20–22). The first observation of myeloid cells with suppressive functions was reported in cancer-bearing mice, where they were able to inhibit T-cell activities (23). The importance of this cell population has been pointed out by accumulating evidence on its contribution to the negative regulation of immune responses during cancer and other diseases in humans. MDSCs are able to inhibit T-cell proliferation and activation (24, 25), modulate cytokine production by macrophages (26), suppress the function of natural killer (NK) cells (27), impair dendritic cell (DC) differentiation and action (28, 29), and induce regulatory T cells (Tregs) (30). Furthermore, MDSCs are capable of inhibiting the proliferation and differentiation of B cells and inducing regulatory B cells in several pathological conditions (31–34).

Besides their immunological functions, MDSCs exert other actions such as the promotion of tumor angiogenesis (35, 36), invasion, and metastasis (37), indicating that they can exert pleiotropic activities.

A pro-inflammatory microenvironment is responsible for MDSC expansion, inducing their proliferation, recruitment, and activation. Several factors, usually associated with pro-inflammatory processes, are involved in MDSC differentiation: prostaglandin E2 (PGE-2) (38), cyclooxygenase-2 (COX-2) (39), stem cell factor (SCF) (40), macrophage colony-stimulating factor (M-CSF), granulocyte/macrophage colony-stimulating factor (GM-CSF) (41), IL-6 (42), tumor necrosis factor alpha (TNF-α) (43), IFN-γ (44), and vascular endothelial grow factor (VEGF) (45). These molecules trigger the STAT3 pathway, which is the master transcription factor regulating the expressions of genes involved in the expansion of MDSCs (46).

## MDSC Identification and Function

MDSCs include two major subsets based on their phenotypic and morphological features: polymorphonuclear (PMN) and monocytic (M) MDSCs. In physiological conditions, bone marrow hematopoietic stem cells (HSCs) differentiate into common myeloid progenitor (CMP) cells and then into immature myeloid cells (IMCs). Normally, IMCs migrate to different peripheral organs, where they differentiate into dendritic cells, macrophages, and granulocytes. However, factors produced by tumor cells or during acute or chronic infections and after trauma promote MDSC generation by preventing IMC differentiation and acquiring immunosuppressive functions (38, 44, 47, 48). The classic definition of MDSCs as immature myeloid cells blocked in their differentiation has been updated by recent studies suggesting that, under certain conditions, M-MDSCs and PMN-MDSCs may originate from monocytes and granulocytes (49). Thus, the family of MDSCs includes not only immature cells but also highly differentiated elements belonging to the monocyte and granulocyte lineages.

Murine PMN-MDSC can be clearly identified as CD11b^++^Ly6G^+^Ly6C^low^ cells, while M-MDSCs as CD11b^+^Ly6G^−^Ly6C^high^. Due to the lack of specific markers for human MDSCs, recently, a standard characterization has been suggested for their identification: among human peripheral blood mononuclear cells (PBMCs), the equivalent to PMN-MDSCs is defined as HLA-DR^−/low^CD11b^+^CD14^−^CD15^+^ (or CD66b^+^) and M-MDSCs as HLA-DR^−/low^CD11b^+^ (or CD33^+^) CD14^+^CD15^−^. A third group of MDSCs, early-stage MDSCs (e-MDSCs), can be identified as HLA-DR^−^CD33^+^CD15^−^Lin^−^ (CD3^−^CD56^−^CD19^−^CD14^−^) (50). The ability to suppress immune cells is an important characteristic of MDSCs, and the gold standard for the designation of cells as MDSCs is the inhibition of T-cell functions (50).

MDSCs have a potent immunosuppressive function that is mediated by different mechanisms: they are able to deplete l-arginine from the microenvironment by secreting arginase 1 Arg-1 and inducible nitric oxide synthase (iNOS). The deprivation of l-arginine inhibits the activity of T cells by decreasing CD3ζ, a key molecule in T-cell receptor (TCR) signaling. Other important factors that contribute to the suppressive activity of MDSCs are reactive oxygen species (ROS) and reactive nitrogen species (RNS). ROS production reduces the expression of CD3ζ on T cells (51), and its inhibition abrogates the suppressive effects of MDSCs *in vitro* (52). The RNS peroxynitrite produced by MDSCs is able to silence T-cell activation by nitrating TCR and CD8 molecules, thus preventing antigen-specific stimulation (25). Furthermore, the production of nitric oxide (NO) by iNOS interferes with the JAK/STAT signaling pathway in T cells (24, 53).

MDSCs can secrete transforming growth factor beta (TGF-β) and IL-10, which exert direct immunosuppressive effects on T cells, induce the generation of Tregs (30), and inhibit IL-12 production by macrophages (26). Moreover, MDSCs can suppress the activity of NK cells by expressing membrane-bound TGF-β (27). Finally, MDSCs express programmed death-ligand 1 (PD-L1), a potent mediator of immunosuppression (54, 55). The engagement of PD-L1 with programmed cell death protein 1 (PD-1) in T cells induces dysfunction, exhaustion, and IL-10 production (56).

## MDSCs and COVID-19

### MDSC Expansion During SARS-CoV-2 Infection

Several reports have highlighted the potential role of MDSCs during infections. In particular, in humans, some bacterial (57), viral (58), and parasitic (59) infections are characterized by the expansion of the MDSC population. The first paper showing the expansion of MDSCs during COVID-19 was published in October 2020 by our group, showing a high frequency of phenotypically resembling MDSCs in PBMCs from patients with COVID-19 (60). MDSCs frequency correlated with the level of inflammatory mediators in patients with COVID-19. We then showed a massive expansion of PMN-MDSCs in severe COVID-19 patients with the capacity to inhibit T-cell proliferation and IFN-γ production upon superantigen stimulation (61). In the same paper, we followed COVID-19 patients after hospital admission and found a persistently higher frequency of PMN-MDSCs in patients with severe compared to those with mild disease.

One of the features of severe COVID-19 is the altered neutrophil abundance, phenotype, and functionality. A high number of neutrophils have been observed in the nasopharyngeal epithelium (62), the lung (63), and in the blood of patients infected with SARS-CoV-2 (64). Interestingly, single-cell RNA sequencing (scRNA-seq) revealed the emergence of CD10^low^CD101^−^CXCR1^+^ immature neutrophils that are reminiscent of PMN-MDSCs (65, 66). Thus, immunosuppressive neutrophil precursors, such as the pre-neutrophil (preNeu) population, which is CXCR4-positive (67), may be released prematurely into the blood from the bone marrow and infiltrate the lung tissue in patients with severe disease. We could then speculate that the expansion of PMN-MDSCs may account, at least in part, for the neutrophilia observed during severe COVID-19.

ScRNA-seq revealed high levels of HLA-DR^−/low^ monocytes in patients with severe COVID-19, whose phenotype resembled M-MDSCs (65, 66, 68). The scRNA-seq data were confirmed by Falck-Jones et al. using flow cytometry, showing an increased frequency of M-MDSCs in the blood of patients with severe COVID-19 (69), even if to a lesser extent than PMN-MDSCs. The highest levels of MDSCs were reported in fatal cases of COVID-19 (69, 70), suggesting a detrimental role of MDSCs in COVID-19. A significant increase of low-density neutrophils (LDNs) expressing lectin-type oxidized low-density lipoprotein receptor 1 (LOX-1) in the blood of patients with acute COVID-19 was also observed (71). Functional assays demonstrated the immunosuppressive capacities of these cells, thus confirming them as PMN-MDSCs. LOX-1 has been recently identified as a specific marker distinguishing PMN-MDSCs from polymorphonuclear cells (PMNs). In fact, among PMNs, the LOX-1^+^ subset exerted a potent suppressive activity (72).

Immunometabolic phenotypical characterization of PBMCs from COVID-19 patients also highlighted the presence of voltage-dependent anion channel (VIDACI)^+^ hexokinase II (HKII)^+^ PMN-MDSCs (73). The concurrent upregulation of VIDACI and HKII has been described to be associated with the production of ROS (74) and with the prevention of ROS-induced cell death (75), aimed, with other mechanisms, at increasing MDSC survival in the presence of high ROS levels (76). Moreover, carnitine palmitoyltransferase 1a (CPT1a)^+^VIDCAI^+^DR^−^ M-MDSC expansion was observed in patients with severe COVID-19 (73). CPT1a is involved in fatty acid oxidation and has been correlated with the recruitment and differentiation of MDSCs (77).

Hyper-inflammation is a hallmark of severe COVID-19 (2). Pro-inflammatory mediators are pivotal in the regulation of MDSC differentiation and accumulation (78). Indeed, during COVID-19, the frequency of MDSC correlated with the plasma levels of IL-1β, IL-6, IL-8, and TNF-α (69–71), confirming that the immune system attempted to curb the excessive and potentially harmful immune response to SARS-CoV-2 infection. However, in severe COVID-19 patients, in addition to monocytes, MDSCs were able to produce IL-6 under stimulation (79), suggesting that they could contribute to hyper-inflammation in certain conditions.

### Suppressive Functions of MDSC During COVID-19

Studies evaluating MDSC function showed that MDSCs from COVID-19 patients were correlated with Arg-1 activity. Several papers have reported high levels of Arg-1 in the plasma of patients with moderate to severe/fatal COVID-19 (69, 80). Accordingly, Reizine et al. found that the expansion of MDSCs was paralleled by a high Arg-1 activity, evaluated as the ornithine/arginine ratio in plasma of patients with ARDS at hospital admission. However, the difference in Arg-1 activity was lost at the subsequent time points (81). The high activity of Arg-1 paralleled with plasma l-arginine shortage (80–82). Importantly, the addition of l-arginine to MDSC/T-cell co-cultures partially restored the production of IFN-γ and the proliferation of T cells (69, 81). Bost et al. obtained similar results, showing a correlation between the percentage of suppression of M-MDSCs and the plasma levels of Arg-1 (83). PMN-MDSCs from COVID-19 patients were able to inhibit the SARS-CoV-2-specific IFN-γ production by T cells (70) and expressed high levels of Arg-1, iNOS, and TGF-β messenger RNAs (mRNAs). However, treatment with a specific inhibitor of Arg-1 was not able to restore IFN-γ production. Differently, an increase of IFN-γ release was observed by inhibiting iNOS or by neutralizing TGF-β (70). Furthermore, a persistently higher indolamine-2,3-dioxygenase (IDO) activity was found in patients with ARDS compared to those with moderate pneumonia (79, 81). IDO catabolizes tryptophan, which is another essential amino acid for T-cell function; indeed, the decrease of tryptophan and the accumulation of its catabolites inhibited the activation of T cells (84).

Altogether, these data indicate that MDSCs from COVID-19 patients exert their suppressive activity using different mechanisms, which possibly depend on the MDSC subsets involved. MDSCs establish different metabolic pathways in different microenvironments, determining different mechanisms of suppression (85). Whether the diverse degrees of severity of COVID-19 could influence the suppressive functions of MDSCs needs further investigation.

MDSCs seem to be able to infiltrate the lung during infection. Immunohistochemistry and immunofluorescence on the lung autopsy of patients who died due to COVID-19 showed the presence of large numbers of Arg-1-positive cells and a high expression of intracytoplasmic Arg-1 in CD66b^+^, confirming them to be Arg-1-positive PMN-MDSC-like cells (80).

It has been demonstrated that MDSCs regulate the immune response of B cells directly by the expression of effector molecules and indirectly by controlling other immune regulatory cells (86). While the suppressive ability of MDSCs on T-cell proliferation and cytokine production has been assessed during COVID-19, their potential action on B-cell function has not been explored. To our knowledge, only one paper evaluated the correlation between the frequency of PMN-MDSCs and the level of anti-SARS-CoV-2 S1 immunoglobulin G (IgG) in 10 convalescence patients who recovered from a mild or asymptomatic SARS-CoV-2 infection. The authors showed an almost significant negative correlation between PMN-MDSCs and anti-S IgG (87), suggesting a possible involvement of MDSCs on B-cell functional modulation. However, a larger group is needed to confirm this preliminary result.

### MDSC and Platelet Activation

We and others found a decrease of plasma l-arginine during severe COVID-19 that correlated with the activities of Arg-1 and iNOS (80–82). We also demonstrated that the frequency of PMN-MDSCs directly correlated with platelet activation, and purified PMN-MDSCs from patients with COVID-19 were able to induce platelet activation, possibly by reducing l-arginine. The concentration of l-arginine can modulate platelet activation and aggregation (88). Indeed, its deprivation reduced substrate availability for iNOS, thus reducing NO production. NO plays a pivotal role in inhibiting platelet activation. It enters the platelets and promotes the upregulation of cyclic guanosine monophosphate (cGMP). cGMP activates protein kinase G, which directly diminishes platelet reactivity by phosphorylating crucial proteins involved in platelet activation (88).

Altogether, these data highlight a novel role of MDSCs in driving the immunopathogenesis of COVID-19 and increasing the complexity of the immune response to SARS-CoV-2.

### MDSCs as a Biomarker of COVID-19 Severity

Studies evaluating the frequency of MDSC-like cells in patients with different COVID-19 disease severities showed the highest MDSC percentages in the severe form of the disease (61, 68–71, 79, 80, 89). In detail, patients with more severe COVID-19 disease had significantly higher percentage of M-MDSCs and PMN-MDSCs compared with those with mild disease and healthy donors (HDs) (61, 69, 70, 80). The frequency of M-MDSCs in blood from patients with COVID-19 seemed to decrease over time and returned to frequency similar to those seen in HDs in follow-up samples taken during convalescence (69). ScRNA-seq revealed the expansion of monocytes with a MDSC-like phenotype only in severe COVID-19 patients (68). The expansion of LDNs with suppressive function was observed in severe COVID-19 patients. In particular, LOX-1^+^ LDNs were higher in severe COVID-19 compared to mild cases (71). Tomic et al. showed increased frequency and number of both M-MDSCs and PMN-MDSCs in the group of patients with severe disease compared to those with mild COVID-19 and in HDs. Moreover, principal component analysis (PCA) showed a clear clustering of severe, mild, and non-COVID-19 disease, suggesting that MDSCs are associated with the severity of COVID-19. Moreover, the frequency of MDSCs producing IL-10 was higher in severe compared to mild COVID-19 patients and in HDs (79). Some studies also observed a higher frequency of MDSCs in non-survival compared to survival patients (69–71), suggesting that the frequency of MDSCs could be used as a predictive marker of the disease outcome. Specifically, a receiver operating characteristic (ROC) curve analysis indicated that the frequency of MDSCs at the time of hospitalization might predict fatal outcomes of the disease, with a cutoff value of 54.91% (70). Moreover, by estimating the hazard ratio (HR) of death adjusted for age and gender, it has been observed that the percentage of PMN-MDSCs at the time of hospitalization was independently associated with fatal outcomes of COVID-19 (70).

Overall, several publications have shown that the MDSC frequency could be used as a biomarker of COVID-19 severity. However, larger prospective multicenter studies are needed to evaluate the predictive biomarker potential of MDSCs and its possible use in monitoring disease progression.

Due to the suppressive role of MDSCs, it would be very interesting to examine whether they could play a role in the response to anti-SARS-CoV-2 vaccinations in fragile patients. Vaccines are the main tools in counteracting SARS-CoV-2-induced disease. Several studies have reported a low humoral response after vaccination in patients with malignancies or diseases that required immunosuppressive therapies (90–94). Different mechanisms can be postulated, such as the underlying disease and, mostly, the treatment of the specific disease. Whether MDSCs can impact on the effectiveness of vaccination is completely unexplored and should be investigated in order to evaluate new markers for patient monitoring and vaccine treatment optimization.

### MDSCs as Therapeutic Targets in COVID-19

**Figure 1:**
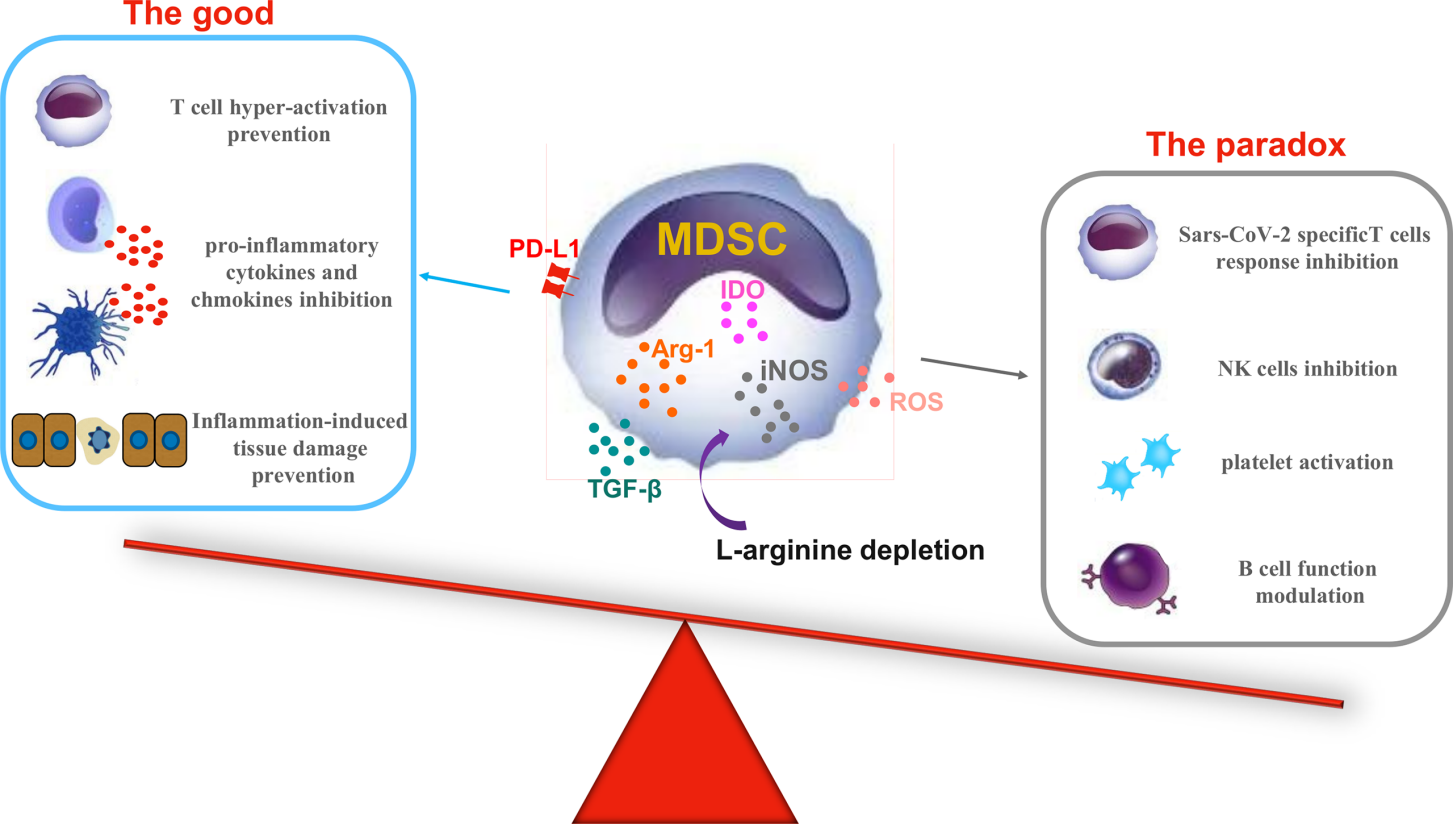
The good and the paradox of myeloid-derived suppressor cell (MDSC) activities during coronavirus disease 2019 (COVID-19). During severe acute respiratory syndrome coronavirus 2 (SARS-CoV-2) infection, MDSC subsets increase and are activated by the virus-induced inflammatory response. The *left panel* shows the plausible beneficial role of suppressive molecules produced by MDSCs during COVID-19 (the good); the *right panel* shows the downside of good, pointing out the detrimental effects of the same molecules (the paradox). *Arg-1*, arginase 1; *IDO*, indoleamine-2,3-dioxygenase; *iNOS*, inducible nitric oxide synthase; *ROS*, reactive oxygen species; *PD-L1*, programmed death-ligand 1; *TGF-β*, transforming growth factor beta.

Data on MDSCs during COVID-19 suggest the detrimental role of this suppressive cell population and highlight the rationale for possible use of therapeutic approaches focused on reducing MDSC number/function. Surprisingly, Bost et al. reported a decline in the percentage of T-cell suppression by monocytes/M-MDSC-like and LDNs/PMN-MDSC cells in severe compared to mild patients. An even lower percentage of T-cell suppression was observed in non-survival patients (83). Altogether, these data raise the question of the balance between the beneficial/detrimental roles of MDSCs during the different stages of COVID-19 (**Figure 1**).

Preclinical studies on new cancer therapy approaches proposed numerous strategies to target MDSCs: i) depletion of MDSCs (95, 96); ii) inhibition of the suppressive functions of MDSCs (97–99); iii) prevention of MDSC recruitment (100, 101); and iv) induction of MDSC differentiation toward monocytes/granulocytes (102, 103).

Among the therapeutic approaches that are being tested to treat COVID-19, some could affect MDSC differentiation and function. The first example is the anti-IL-6 receptor tocilizumab. As mentioned above, due to the association between the plasma levels of IL-6 and the fatal outcomes of COVID-19, the anti-IL-6 receptor monoclonal antibody tocilizumab was introduced for treatment of the disease, showing reduced mortality, an increased chance of successful hospital discharge, and a reduced risk of invasive mechanical ventilation (104). IL-6 inhibitors have been successfully tested for their ability to block MDSC expansion (105). However, the impact of tocilizumab, or other immunomodulatory agents, on MDSC frequency has been poorly explored. Tomić et al. did not find any difference in the frequency of M-MDSCs and PMN-MDSCs in a very small group of COVID-19 patients previously treated with tocilizumab compared to non-treated patients matched for age, sex, and disease severity (79). New studies are necessary to clarify the effect of this inhibitor on the modulation of the number and function of MDSCs during treatment and its association with therapy efficacy.

Some clinical trials are ongoing evaluating the effect of l-arginine supplementation on clinical improvements of COVID-19 (clinicaltrials.gov). l-Arginine is involved in several biological processes, including the regulation of endothelial function, serving as a substrate for NO production by endothelial cells, thus regulating cardiovascular homeostasis (106). MDSCs exert their suppressive functions through Arg-1 and iNOS, which are involved in reducing the availability of l-arginine during COVID-19 (107). *In vitro* l-arginine addition increased the proliferation rates of CD4 and CD8 T cells from COVID-19 patients (81). It would be interesting to evaluate whether *in vivo* l-arginine supplementation may overcome the MDSC-mediated l-arginine deprivation.

A few studies also suggested an association between vitamin D deficiency and hospitalization risk or COVID-19 severity (108, 109), and several trials are evaluating the impact of vitamin D treatment on the outcomes of COVID-19. Vitamin D has been demonstrated to inhibit the expansion and suppressive functions of MDSC (110, 111), but studies evaluating the correlation between vitamin D levels and MDSC functions during COVID-19 are still lacking.

Another example is leronlimab, an inhibitor of CCR5 signaling. Preliminary studies have shown that leronlimab treatment of COVID-19 patients induced a reduction of plasma IL-6, restoration of the CD4/CD8 ratio, and resolution of SARS-CoV-2 plasma viremia (112). CCR5 has a critical role not only in the recruitment but also in the activation of MDSCs in tumor lesions (113), and targeting the CCR5/CCL5 axis may reduce the suppressive activity of MDSCs (114).

Many other molecules have been successfully tested in cancer settings to reduce the accumulation and suppressive functions of MDSCs. Some of these had STAT3 signaling as a target: sunitinib reversed tumor MDSC accumulation through STAT3 or c-Kit signaling, and metformin downregulated the function of MDSCs through the AMPK/STAT3 pathway. Entinostat, a class I histone deacetylase inhibitor, neutralized MDSCs through reducing the expressions of both Arg-1 and iNOS [reviewed in (115)]. Another molecule tested is all-*trans* retinoic acid (ATRA). ATRA is a standard component of therapy for patients with acute promyelocytic leukemia (116), and several reports indicated its efficacy in reducing the number of MDSCs in murine cancer models (96, 117, 118). ATRA acts by inducing the differentiation of MDSCs toward monocytes, DCs, or granulocytes, thus abolishing their suppressive functions (119, 120). Interestingly, ATRA has shown an anti-SARS-CoV-2 activity *in vitro* (121, 122), possibly by increasing the expression of retinoic acid-inducible gene I (RIG-I) on target cells (123).

Altogether, these data suggest the possibility of combining immunomodulatory treatments and new approaches targeting MDSCs to avoid the detrimental role of MDSCs on SARS-CoV-2-specific immunity and platelet activation and, at the same time, to reduce the harmful impacts of the inflammatory storm and viral replication.

## Conclusion

SARS-CoV-2 infection induces massive activation of the immune system, ultimately leading to the suppression of innate and adaptive immune responses. MDSCs are key cellular players in this complicated process. Both M-MDSCs and PMN-MDSCs accumulate in patients with COVID-19 and represent an attempt to maintaining a homeostatic balance between protective immune response and a maladaptive damaging uncontrolled inflammation. However, MDSC accumulation associates with fatal disease outcomes, indicating the detrimental role of MDSCs possibly mediated by the suppression of the adaptive immune response. More investigations are essential to define when (in terms of time or percentage) the suppressive functions of MDSCs could be harmful rather than beneficial during COVID-19. Clarifying this issue is pivotal to evaluating the timing and type of possible therapeutic approaches targeting MDSCs.

## Author Contributions

AS, GG, and CA wrote sections of the paper. All authors contributed to manuscript revision, read, and approved the submitted version.

## Funding

This work was funded by Ministry of Health (Ricerca Corrente LR 1).

## Conflict of Interest

The authors declare that the research was conducted in the absence of any commercial or financial relationships that could be construed as a potential conflict of interest.

## Publisher’s Note

All claims expressed in this article are solely those of the authors and do not necessarily represent those of their affiliated organizations, or those of the publisher, the editors and the reviewers. Any product that may be evaluated in this article, or claim that may be made by its manufacturer, is not guaranteed or endorsed by the publisher.
